# Registered report: Coding-independent regulation of the tumor suppressor PTEN by competing endogenous mRNAs

**DOI:** 10.7554/eLife.12470

**Published:** 2016-03-01

**Authors:** Mitch Phelps, Chris Coss, Hongyan Wang, Matthew Cook, Reproducibility Project: Cancer Biology, Elizabeth Iorns

**Affiliations:** Science Exchange, Palo Alto, United States; Mendeley, London, United Kingdom; Science Exchange, Palo Alto, United States; Science Exchange, Palo Alto, United States; Science Exchange, Palo Alto, United States; Center for Open Science, Charlottesville, United States; 1 Pharmacoanalytic Shared Resource, The Ohio State University, Columbus, United States; 2 University of California San Francisco, San Francisco, United States; Case Western Reserve University, United States

**Keywords:** reproducibility project: cancer biology, methodology, microRNA, PTEN, Human

## Abstract

The Reproducibility Project: Cancer Biology seeks to address growing concerns about reproducibility in scientific research by conducting replications of selected experiments from a number of high-profile papers in the field of cancer biology. The papers, which were published between 2010 and 2012, were selected on the basis of citations and Altmetric scores (Errington et al., 2014). This Registered Report describes the proposed replication plan of key experiments from “Coding-Independent Regulation of the Tumor Suppressor PTEN by Competing Endogenous 'mRNAs' by Tay and colleagues, published in *Cell* in 2011 (Tay et al., 2011). The experiments to be replicated are those reported in Figures 3C, 3D, 3G, 3H, 5A and 5B, and in Supplemental Figures 3A and B. Tay and colleagues proposed a new regulatory mechanism based on competing endogenous RNAs (ceRNAs), which regulate target genes by competitive binding of shared microRNAs. They test their model by identifying and confirming ceRNAs that target *PTEN*. In Figure 3A and B, they report that perturbing expression of putative *PTEN* ceRNAs affects expression of PTEN. This effect is dependent on functional microRNA machinery (Figure 3G and H), and affects the pathway downstream of PTEN itself (Figures 5A and B). The Reproducibility Project: Cancer Biology is a collaboration between the Center for Open Science and Science Exchange, and the results of the replications will be published by *eLife*.

**DOI:** http://dx.doi.org/10.7554/eLife.12470.001

## Introduction

microRNAs are one of the first identified classes of non-coding RNAs that can modulate the expression of mRNA-coding transcripts by binding to complementary regions in a target gene’s sequence and repressing its expression. Thus, expression levels and availability of these microRNAs can influence gene expression, and there is growing evidence that misregulation of microRNAs is correlated with some forms of cancer (Sen et al., 2014). Naturally occurring microRNA 'sponges' have been shown to be effective in regulating gene expression by altering the levels of their cognate microRNAs (Choi et al., 2007; Karreth and Pandolfi 2013). Poliseno and colleagues proposed that pseudogenes, long non-coding RNAs with strong homology to coding sequences, could act as the modulators of gene expression as microRNA sponges (Poliseno et al., 2010). They demonstrated that the pseudogene *PTENP1* could regulate the expression levels of *PTEN* via their cognate microRNAs miR-19b and miR-20a (Poliseno et al., 2010).

In this study, Tay and colleagues expanded upon the previous work to propose a unifying hypothesis of regulatory networks composed of competing endogenous RNAs (ceRNAs) (Karreth and Pandolfi 2013; Sen et al., 2014; Kartha and Subramanian, 2014). They suggest that protein-coding RNAs, not just non-coding RNAs, can cross-regulate each other based on competition for shared microRNA regulators; ceRNAs can titrate microRNAs from their target genes (Tay et al., 2011). Continuing their focus on the regulation of *PTEN*, one of the most frequently mutated genes in cancer (Song et al., 2012), Tay and colleagues propose a computational model to identify ceRNAs de novo, termed MuTaME. Using MuTaME, they identified potential ceRNA regulators of *PTEN*, and validated if these candidate ceRNAs could modulate *PTEN* expression in a microRNA-dependent manner (Tay et al., 2011).

In Figure 3C, Tay and colleagues examine if silencing ceRNAs targeting *PTEN* would affect the expression levels of a luciferase construct carrying the 3’UTR of *PTEN*. They co-transfected DU145 cells with siRNAs against the candidate *PTEN* ceRNAs along with a luciferase-*PTEN 3’UTR* construct and measured luciferase activity. After confirming knockdown of each target ceRNA (Supplemental Figure 3A), they reported that the loss of three of their candidate ceRNAs - *SERINC1, VAPA* and *CNOT6L*, but not *ZNF460* - reduced the luciferase activity of the *PTEN 3’UTR* construct. This experiment will be replicated in Protocol 1.

In Figure 3D, they demonstrated that only the 3’UTRs of the candidate ceRNAs were required to affect changes in the luciferase activity of the *PTEN* 3’UTR construct. Ectopic overexpression of the 3’UTRs of the three candidate ceRNAs relieved inhibition of the *PTEN* 3’UTR, as evidenced by increased luciferase activity as compared to controls. This experiment will be replicated in Protocol 2.

To test if this effect was dependent on microRNAs, Tay and colleagues repeated these experiments in *DICER1* mutant HCT116 cells, in which the machinery required for microRNA function is abrogated. Transfection of wild type HCT116 cells with siRNAs targeting the candidate ceRNAs showed a marked reduction in PTEN protein levels, an effect that was not seen in the *DICER1* mutant HCT116 cells (Figures 3G and H). Knockdown of the candidate ceRNAs was confirmed by RT-PCR (Supplemental Figure 3B). This experiment will be replicated in Protocol 3.

PTEN negatively regulates the PI3K/AKT pathway (Stambolic et al., 1998), so Tay and colleagues examined if ceRNA modulation affected the phosphorylation of AKT. Loss of *CNOT6L* and *VAPA* in DU145 cells elevated pAKT levels after serum stimulation, an effect that was also observed in wild-type HCT116 cells (Figure 5A). However, this effect was abrogated in *DICER1* mutant HCT116 cells (Figure 5A). They also examined the effect of ceRNAs on the tumorigenic properties conferred by loss of PTEN. Silencing of the ceRNAs *CNOT6L* and *VAPA* increased cell proliferation of DU145 cells and wild-type HCT116 cells, similar to silencing of *PTEN* directly (Figure 5B). This effect was less pronounced in *DICER1* mutant HCT116 cells (Figure 5B). These experiments will be replicated in Protocol 4 and 5.

Two papers published simultaneously provide support for the actions of ceRNA regulatory networks. Karreth and colleagues, from the same lab as Tay and colleagues, demonstrated in vivo evidence for the actions of ceRNA regulation using the sleeping beauty transposase system in a mouse model of melanoma to identify and confirm putative *PTEN* ceRNAs (Karreth et al., 2011). Karreth and colleagues identified *CNOT6L* as a putative *PTEN* ceRNA through the sleeping beauty transposase system, providing further evidence that *CNOT6L* is indeed involved in *PTEN* regulation. Karreth and colleagues focused on *ZEB2*; using siRNA silencing, they reported that the loss of *ZEB2* reduced PTEN protein levels, and affected downstream phosphorylation of AKT (Karreth et al., 2011). As seen in Tay and colleagues, these effects were dependent on functional microRNA processing; *ZEB2* depletion did not affect PTEN levels in *DICER1* mutant HCT116 cells (Karreth et al., 2011). Sumazin and colleagues used a bioinformatics approach to identify post-translational regulation and elucidated over 7,000 genes they proposed acted as miRNA sponges. By comparing the miRNA programs of genes, they could identify genes with common miR programs, indicating the potential for miRNA-mediated crosstalk between those two genes (Sumazin et al., 2011). They tested their findings by exploring the regulation of *PTEN*, demonstrating that silencing of putative miRNA program-mediated regulators (mPRs) of *PTEN* decreased *PTEN* expression, and, conversely, that the perturbation of PTEN levels could inversely affect the expression of its mPRs. These manipulations also affected tumor cell growth rates, indicating potential in vivo effects of changes to mPR regulatory networks (Sumazin et al., 2011). Since the publication of these three papers, numerous other examples of ceRNA regulation have been reported in muscle differentiation (Cesana et al., 2011), human embryonic stem cell renewal (Wang et al., 2013), regulation of sex determination by SRY (Granados-Riveron and Aquino-Jarquin 2014), breast cancer (Yang et al., 2014; Zheng et al., 2015a; 2015b), lymphoma (Karreth et al., 2015) and the regulation of the tumor-related HMGA1 (Esposito et al., 2014).

The Pandolfi group followed up on their 2011 paper by generating a mathematical model to predict optimal conditions for ceRNA activity, based on a molecular titration mechanism whose effects were correlated to the relative levels of the ceRNA and its target (Ala et al., 2013). They then tested their in silico predictions by experimentally exploring the effect of *VAPA* on *PTEN* expression. While silencing of *VAPA* did decrease *PTEN* expression in all five cell lines tested, they noted that the amount of silencing was correlated with the initial *VAPA:PTEN* expression ratio (Ala et al., 2013). However, Denzler and colleagues challenge the notion that perturbations in ceRNA expression levels could affect target genes at all (Denzler et al., 2014). Denzler and colleagues and Ala and colleagues both state that ceRNA effects are dependent on the kinetics of binding, which in turn relies upon the ratio of microRNAs to target sites; increasing the number of target sites through expression of ceRNAs is postulated to affect target gene repression. By quantifying the absolute copy number of the well-studied highly abundant miR-122 and its target sites, Denzler and colleagues showed that large, physiologically unlikely changes in ceRNA expression levels would be required to alter the microRNA: target site ratio enough to perturb target gene expression, casting doubt on the ability of these putative ceRNAs to affect changes in target gene expression levels (Broderick and Zamore 2014; Denzler et al., 2014). This view was contradicted by Bosson and colleagues, who identified over 3,000 high affinity target sites they claimed could be affected by ceRNAs due to low endogenous microRNA: target site ratios (Bosson et al., 2014). The activity and impact of potential ceRNA networks is an area of active interest (for review, see de Giorgio et al., 2013).

## Materials and methods

Unless otherwise noted, all protocol information was derived from the original paper, references from the original paper, or information obtained directly from the authors. An asterisk (*) indicates data or information provided by the Reproducibility Project: Cancer Biology core team. A hashtag (#) indicates information provided by the replicating lab.

### Protocol 1: Knock-down of ceRNA network genes results in decreased PTEN-3’UTR luciferase expression

This protocol describes how to silence expression of ceRNA network genes and measure effects on *PTEN* expression by measuring *PTEN* 3’UTR luciferase activity, as seen in Figures 3C and Supplementary S3A.

#### Sampling

- This experiment will include four biological replicates (Luciferase assay) and four biological replicates (qRT-PCR) for a minimum power of 80%.
  - See Power Calculations section for details.
- Each experiment consists of DU145 cells co-transfected with a luciferase-*PTEN* 3’UTR reporter construct and siRNA against *PTEN* ceRNAs:
  - Cohort 1: siRNA against nontargeting control 2 (siNC)
  - Cohort 2: siGENOME siRNA against *SERINC1* (siSER)
  - Cohort 3: siGENOME siRNA against *ZNF460* (siZNF)
  - Cohort 4: siGENOME siRNA against *VAPA* (siVAPA)
  - Cohort 5: siGENOME siRNA against *CNOT6L* (siCNO)
  - Cohort 6: siGENOME siRNA against *PTEN* (siPTEN)
  - Cohort 7: siGLO RISC-free siRNA (transfection control)
- Effects of silencing ceRNAs will be tested with
  - Luciferase assay of *PTEN* 3’UTR expression (Figure 3C)
  - qRT-PCR to confirm target gene silencing (Supplementary Fig S3A)
  - siGLO fluorescence to confirm transfection efficiency

#### Materials and reagents

**Table:** 

Reagent	Type	Manufacturer	Catalog #	Comments
DU145 human prostate cancer cells	Cells	ATCC	HTB-81	
psiCHECK-2-PTEN 3'UTR plasmid	Plasmid	Addgene	plasmid #50936	Communicated by original authors
siGLO RISC-free siRNA siGLO	siRNA	Dharmacon	D-001600-01-05	Catalog # communicated by original authors
siGenome siRNA for nontargeting control 2	siRNA	Dharmacon	D-001210-02-05	Catalog # communicated by original authors
siGenome siRNA for *SERINC1*	siRNA	Dharmacon	M-010725-00-0005	Catalog # communicated by original authors
siGenome siRNA for *ZNF460*	siRNA	Dharmacon	M-032012-01-0005	Catalog # communicated by original authors
siGenome siRNA for *VAPA*	siRNA	Dharmacon	M-021382-01-0005	Catalog # communicated by original authors
siGenome siRNA for *CNOT6L*	siRNA	Dharmacon	M-016411-01-0005	Catalog # communicated by original authors
siGenome siRNA for *PTEN*	siRNA	Dharmacon	M-003023-02-0005	Catalog # communicated by original authors
Dulbecco's Modified Eagle's Medium (DMEM)	Cell Culture Reagent	Invitrogen	10313-039	Catalog # communicated by original authors
Fetal Bovine Serum (FBS)	Cell Culture Reagent	Invitrogen	10438-026	Catalog # communicated by original authors
Penicillin/Streptomycin	Cell Culture Reagent	Life Technologies	15140-163	Communicated by original authors
Glutamine	Cell Culture Reagent	Life Technologies	25030-081	Communicated by original authors
Lipofectamine 2000	Transfection Reagent	Life Technologies	11668500	Communicated by original authors
Trypsin	Transfection Reagent	Life Technologies	15400-054	Communicated by original authors
Dual Luciferase Reporter Assay	Luciferase Assay	Promega	E1960	Catalog # communicated by original authors
Lysis Buffer (included with Dual-Luciferase Reporter Assay)	Buffer	Promega	E1960	Original not specified
GLOMAX 96 Microplate Luminometer	Equipment	Promega	E6501	Replaces Promega E8032 (communicated by original authors)
TRIzol reagent	qPCR reagent	Life Technologies	15596026	Communicated by original authors
RNeasy kit	qPCR reagent	Qiagen	74104	Communicated by original authors
High Capacity cDNA Archive kit	qPCR reagent	Life Technologies	4368814	Communicated by original authors
TaqMan probe *PTEN*	qPCR probes	Life Technologies	Hs02621230_s1	
TaqMan probe *CNOT6L*	qPCR probes	Life Technologies	Hs00375913_m1	
TaqMan probe *VAPA*	qPCR probes	Life Technologies	Hs00427749_m1	
TaqMan probe *SERINC1*	qPCR probes	Life Technologies	Hs00380375_m1	
TaqMan probe *ZNF460*	qPCR probes	Life Technologies	Hs01104252_m1	
TaqMan control probe *ß-ACTIN*	qPCR probes	Life Technologies	Hs00969077_m1	Communicated by original authors
TaqMan Fast Advanced Master Mix	qPCR reagent	Life Technologies	4444964	Communicated by original authors
StepOne Plus Real-Time PCR system	Equipment	Applied Biosystems		Replaces LightCycler 480 System
Nanodrop 2000C Spectrometer	Equipment	Thermo Scientific		

#### Procedure

Notes:

- All cells will be sent for mycoplasma testing and STR profiling.
- DU145 cells are maintained in DMEM supplemented with 10% FBS, ^#^100 U/ml penicillin/100 µg/ml streptomycin, and ^#^2 mM glutamine at 37°C in 5% CO_2_ in a humidified atmosphere.

1. Co-transfect DU145 cells with *PTEN* 3’UTR and siRNAs:
  - Split DU145 cells into four different cultures.
    - These will be biological replicates.
  - Seed cells at 1.2 x 10^5^ cells per well in 12 well dishes for 24.
    - Seed 13 wells: 6 transfection conditions x 2 wells per condition (for Steps 2 and 3) and 1 transfection condition (siGlo RISC free siRNA transfection control) x 1 well.
  - Prepare separate transfection mixtures for each biological replicate.
  - Add 100 ng of psiCHECK-2+PTEN3’UTR and 100 pmol of siRNA QS to 100 µl of Opti-MEM.
    - Transfect a pair of wells with each of the following:
      1. siSERINC1
      2. siZNF460
      3. siVAPA
      4. siCNOT6L
      5. siPTEN
      6. non-targeting control (NC)
    - Transfect a single well with the following:
      1. siGLO control siRNA
  - In a separate tube mix 2 µl of Lipofectamine 2000 with 100 µl of Opti-MEM.
    - Scale the volume according to number of replicates.
    - Incubate for 10 min.
  - Combine the plasmid/siRNA and Lipofectamine mixes with gentle mixing and incubate for an additional 20min.
  - Aliquot 200 µl of the plasmid/siRNA and Lipofectamine transfection mix into appropriate well.
    - Mix gently and incubate at 37˚C.
    - Replace growth medium after 4.
    - After 24-48, count the number of fluorescent cells transfected with siGLO relative to total to confirm >90% transfection efficiency.
      1. If transfection is less than 90%, record efficiency, exclude replicate and omit it from the rest of the procedure. Repeat procedure until >90% efficiency is obtained.
      2. If modification to transfection is needed, record and maintain modified steps for remaining replicates.
  - Incubate for 72 at 37˚C in 5% CO_2_ in a humidified atmosphere
2. Use one well for each transfection to measure luciferase activity:
  - Wash cells with ice-cold PBS, aspirate and add 100 µl of 1X lysis buffer.
  - Place on an orbital shaker for 10min to dissociate the cell layer.
  - Pipette gently to mix and transfer 20 µl of each lysate into one well of a white-walled 96 well plate.
  - Measure firefly and Renilla luciferase activities with the dual-luciferase reporter system with a luminometer according to the manufacturer’s instructions.
3. Using the other well for each transfection, confirm siRNA target knock-down with qRT-PCR:
  - Extract total RNA using TRIzol reagent according to manufacturer’s instructions.
  - Purify samples with RNeasy kit according to manufacturer’s instructions.
  - Quality check RNA by measuring A_260/280_ and A_260/230_ absorbance ratios.
  - Total RNA can be frozen here until all biological replicates are performed after which the remaining steps will be conducted at one time.
  - Reverse transcribe 1 µg total RNA using High Capacity cDNA Archive kit according to manufacturer’s instructions.
  - Perform qRT-PCR to confirm mRNA expression knockdown. Measure mRNA expression for each siRNA transfection sample with its appropriate target and *ß-ACTIN*, and test each probe separately using RNA from the NC transfection.
    - PTEN
    - CNOT6L
    - VAPA
    - ZNF460
    - SERINC1
    - *β-ACTIN* [endogenous control communicated by original author]
      - Prepare 10 µl real-time PCR reaction in triplicate for each reaction consisting of:
    - 5 µl TaqMan mastermix
    - 0.5 µl TaqMan probe for the gene of interest
    - 4.5 µl cDNA (diluted 10x)
    - Use standard TaqMan cycling protocol:
      1. 50˚C 2 min
      2. 95˚C 20 s
      3. 40 cycles of 95˚C 1 s, 60˚C 20 s
  - Normalize each mRNA expression to *ß-ACTIN* and then normalize each siRNA to siNC for that transcript.
4. Repeat steps 1-3, 3 additional times

#### Deliverables

- Data to be collected:
  - QC image data confirming transfection efficiency by measuring the number of fluorescent cells transfected with siGLO
  - Raw data of Renilla and firefly luciferase measures and a graph of luciferase activity for each cohort
  - QC data for total RNA (A_260/280_ and A_260/230_ absorbance ratios)qRT-PCR data to confirm silencing: raw qPCR data and for each sample and a graph of each target gene normalized with *β-ACTIN* and normalized relative to NC expression

#### Confirmatory analysis plan

- Statistical Analysis of the Replication Data:Note: At the time of analysis, we will perform the Shapiro-Wilk test and generate a quantile-quantile plot to assess the normality of the data. We will also perform Levene’s test to assess homoscedasticity. If the data appear skewed, we will perform the appropriate transformation to proceed with the proposed statistical analysis. If this is not possible, we will perform the equivalent non-parametric Wilcoxon-Mann-Whitney test.
  - Luciferase assay: One-way ANOVA of luciferase activity in DU145 cells transfected with siRNA against NC, *SERINC1, ZNF460, VAPA, CNOT6L*, or *PTEN*, with the following Bonferroni-corrected comparisons:
    - Non-coding siRNA vs. each of the ceRNA transfected cells (5 comparisons total).
  - qRT-PCR: Bonferroni corrected one-sample *t*-tests of normalized mRNA expression in DU145 cells transfected with siRNA against *SERINC1, ZNF460, VAPA, CNOT6L*, or *PTEN* compared to a constant (siNC = 1) (5 comparisons total).
- Meta-analysis of original and replication attempt effect sizes:
  - This replication attempt will perform the statistical analysis listed above, compute the effects sizes, compare them against the reported effect size in the original paper, and use a meta-analytic approach to combine the original and replication effects, which will be presented as a forest plot.

#### Known differences from the original study

All known differences are listed in the materials and reagents section above with the originally used item listed in the comments section. All differences have the same capabilities as the original and are not expected to alter the experimental design.

#### Provisions for quality control

Extracted RNA integrity will be reported with A_260/280_ and A_260/230_ absorbance ratios, and transfection efficiency will be checked using the siGLO control. qRT-PCR will be performed to confirm the silencing of ceRNA expression. The cells will be sent for mycoplasma testing confirming lack of contamination and STR profiling confirming cell line authenticity. Transfection efficiency will be recorded for each replicate and any transfection that does not contain >90% efficiency will be excluded and not continue through the rest of the procedure. Any modifications to the transfection protocol will be recorded, and the procedure will be maintained for the remaining replicates. All data obtained from the experiment - raw data, data analysis, control data and quality control data - will be made publicly available, either in the published manuscript or as an open access dataset available on the Open Science Framework (https://osf.io/oblj1/).

### Protocol 2: Overexpression of PTEN ceRNA 3’UTRs network genes results in upregulation of PTEN3’UTR luciferase activity

This protocol describes how to measure the effect of ectopic overexpression of PTEN ceRNA 3’UTRs in DU145 cells on Luc-PTEN 3’UTR levels. This protocol replicates Figures 3D.

#### Sampling

- This experiment will include six biological replicates for a minimum power of 88%.
  - See Power calculations for details.
- Each experiment consists of DU145 cells co-transfected with a luciferase-PTEN 3’UTR reporter construct and:
  - Cohort 1: *SERINC1* 3’UTR (SER 3’U)
  - Cohort 2: *VAPA* 3’UTR1 (VAPA 3’U1)
  - Cohort 3: *VAPA* 3’UTR2 (VAPA 3’U2)
  - Cohort 4: *CNOT6L* 3’UTR1 (CNO 3’U1)
  - Cohort 5: *CNOT6L* 3’UTR2 (CNO 3’U2)
  - Cohort 6: *PTEN* 3’UTR (PTEN 3’U)
  - Cohort 7: Empty vector control
- Effects of overexpressing ceRNAs will be tested with
  - Luciferase assay of PTEN 3’UTR expression (Figure 3D)

#### Materials and reagents

**Table:** 

Reagent	Type	Manufacturer	Catalog #	Comments
DU145 human prostate cancer cells	Cells	ATCC	HTB-81	
psiCHECK-2-PTEN 3'UTR plasmid	Plasmid	Addgene	plasmid #50936	Communicated by original authors
psiCHECK-2 empty vector	Plasmid	Promega	C8021	Catalog # communicated by original authors
Dulbecco's Modified Eagle's Medium (DMEM)	Cell Culture Reagent	Invitrogen	10313-039	Catalog # communicated by original authors
Fetal Bovine Serum (FBS)	Cell Culture Reagent	Invitrogen	10438-026	Catalog # communicated by original authors
Penicillin/Streptomycin	Cell Culture Reagent	Life Technologies	15140-163	Communicated by original authors
Glutamine	Cell Culture Reagent	Life Technologies	25030-081	Communicated by original authors
Lipofectamine 2000	Transfection Reagent	Life Technologies	11668500	Communicated by original authors
*SERINC1* 3’UTR vector	Plasmid	Provided by original authors		
*VAPA* 3’UTR1 vector	Plasmid	Provided by original authors		
*VAPA* 3’UTR2 vector	Plasmid	Provided by original authors		
*CNOT6L* 3’UTR1 vector	Plasmid	Provided by original authors		
*CNOT6L* 3’UTR2 vector	Plasmid	Provided by original authors		
*PTEN* 3’UTR vector	Plasmid	Provided by original authors		
Trypsin	Transfection Reagent	Life Technologies	15400-054	Communicated by original authors
Dual Luciferase Reporter Assay	Luciferase Assay	Promega	E1960	Catalog # communicated by original authors
Luminometer	Equipment	Promega	E8032	Catalog # communicated by original authors

#### Procedure

Notes:

- All cells will be sent for mycoplasma testing and STR profiling.
- DU145 cells are maintained in DMEM supplemented with 10% FBS, ^#^100 U/ml penicillin/100 μg/ml streptomycin, and ^#^2 mM glutamine at 37˚C in 5% CO_2_ in a humidified atmosphere.

1. Transfect DU145 cells with *PTEN* 3’UTR and ceRNA 3’UTRs:
  - Separate DU145 cells into 6 different cultures.
    - These will be biological replicates.
  - Seed cells at 1.2 x 10^5^ cells per well in 12 well dishes and incubate for 24 hr.
    - Seed 1 well per biological replicate: 7 transfections x 6 replicates.
      1. 42 wells total seeded.
  - Prepare the transfection mix by adding 100 ng of psiCHECK-2+PTEN3’UTR and 1 µg of 3’UTR plasmid to 100 µl of Opti-MEM.
    - Transfect one well per replicate with each of the following:
      1. *SER* 3’U
      2. *VAPA* 3’U1
      3. *VAPA* 3’U2
      4. *CNO* 3’U1
      5. *CNO* 3’U2
      6. *PTEN* 3’U
      7. empty vector control
  - In a separate tube, mix 2 µl of Lipofectamine 2000 with 100 µl of Opti-MEM.
    - Scale the volume of reagents accordingly.
    - Incubate for 10 min.
  - Combine the plasmid and Lipofectamine mixes and incubate for an additional 20 min.
  - Aliquot 200 µl of the plasmid and Lipofectamine transfection mix into each well. Mix gently and incubate at 37˚C in 5% CO_2_ in a humidified atmosphere.
    - Replace growth medium after 4 hr.
  - Incubate for 72 hr.
2. Measure renilla and firefly luciferase activity as outlined in Protocol 1 Step 2.

#### Deliverables

- Data to be collected:
  - Raw data of Renilla and firefly luciferase measures and a graph of luciferase activity for each cohort.

#### Confirmatory analysis plan

- Statistical Analysis of the Replication Data:Note: At the time of analysis, we will test for normality and homoscedasticity of the data. If the data appears skewed, we will perform the appropriate transformation to proceed with the proposed statistical analysis. If this is not possible, we will perform the equivalent non-parametric Wilcoxon-Mann-Whitney test.
  - One-way ANOVA of luciferase activity in DU145 cells expressing 3’UTRs *SER, VAPA* 3’U1, *VAPA* 3’U2, *CNO* 3’U1, *CNO* 3’U2, *PTEN*, or empty vector control with the following Bonferroni-corrected planned comparisons:
    - Luciferase activity in each 3’UTR transfection vs. the empty vector control (6 comparisons total).
- Meta-analysis of original and replication attempt effect sizes:
  - This replication attempt will perform the statistical analysis listed above, compute the effects sizes, compare them against the reported effect size in the original paper and use a meta-analytic approach to combine the original and replication effects, which will be presented as a forest plot.

#### Known differences from the original study

All known differences are listed in the materials and reagents section above with the originally used item listed in the comments section. All differences have the same capabilities as the original and are not expected to alter the experimental design.

#### Provisions for quality control

The cells will be sent for mycoplasma testing confirming lack of contamination and STR profiling confirming cell line authenticity. All data obtained from the experiment - raw data, data analysis, control data and quality control data - will be made publicly available, either in the published manuscript or as an open access dataset available on the Open Science Framework (https://osf.io/oblj1/).

### Protocol 3: Knock-down of ceRNA network genes results in decreased PTEN protein that is dependent on microRNA functioning

This protocol describes how to test the effects of siRNA-mediated depletion of *SERINC1, VAPA*, or *CNOT6L* expression on PTEN protein expression in wild-type HCT116 colon cancer cells. It also tests whether these effects are dependent on mature microRNA using Dicer mutant (DICER^Ex5^) HCT116 cells. It replicates Figures 3G,H, and Supplementary Figure 3B.

#### Sampling

- The experiment will be repeated four times (Western blot) and three times (qRT-PCR) for a minimum power of 80%.
  - See Power Calculations section for details.
- Each experiment consists of HCT116 WT and HCT116 DICER^Ex5^ cells transfected with siRNA against PTEN ceRNAs:
  - Cohort 1: siRNA against nontargeting control 2 (siNC)
  - Cohort 2: siGENOME siRNA against *SERINC1* (siSER)
  - Cohort 3: siGENOME siRNA against *VAPA* (siVAPA)
  - Cohort 4: siGENOME siRNA against *CNOT6L* (siCNO)
  - Cohort 5: siGENOME siRNA against *PTEN* (siPTEN)
  - Cohort 6: siGLO RISC-free siRNA (siGLO)
- Effects of silencing ceRNAs will be tested with
  - Western Blot for PTEN protein (Figure 3G & 3H)
  - qRT-PCR to confirm target genes were silenced (Supplementary Figure 3B)
  - siGLO fluorescence cell counts to confirm transfection efficiency

#### Materials and reagents

**Table:** 

Reagent	Type	Manufacturer	Catalog #	Comments	
HCT116 WT and DICER^Ex5^ cells	Cells	Horizon Discovery	HD R02-019		
siGLO RISC-free siRNA siGLO	siRNA	Dharmacon	D-001600-01-05		
siGenome siRNA for nontargeting control 2	siRNA	Dharmacon	D-001210-02-05	Catalog # communicated by original authors	
siGenome siRNA for *SERINC1*	siRNA	Dharmacon	M-010725-00-0005	Catalog # communicated by original authors	
siGenome siRNA for *VAPA*	siRNA	Dharmacon	M-021382-01-0005	Catalog # communicated by original authors	
siGenome siRNA for *CNOT6L*	siRNA	Dharmacon	M-016411-01-0005	Catalog # communicated by original authors	
siGenome siRNA for *PTEN*	siRNA	Dharmacon	M-003023-02-0005	Catalog # communicated by original authors	
Dulbecco's Modified Eagle's Medium (DMEM)	Cell Culture Reagent	Invitrogen	10313-039	Catalog # communicated by original authors	
Fetal Bovine Serum (FBS)	Cell Culture Reagent	Invitrogen	10438-026	Catalog # communicated by original authors	
Penicillin/Streptomycin	Cell Culture Reagent	Life Technologies	15140-163	Communicated by original authors	
Glutamine	Cell Culture Reagent	Life Technologies	25030-081	Communicated by original authors	
Trypsin	Transfection Reagent	Life Technologies	15400-054	Communicated by original authors	
Dharmafect 1	Transfection Reagent	Thermo Fisher Scientific	T200104	Communicated by original authors	
TRIzol reagent	qPCR reagent	Life Technologies	15596026	Communicated by original authors	
RNeasy kit	qPCR reagent	Qiagen	74104	Communicated by original authors	
High Capacity cDNA Archive kit	qPCR reagent	Life Technologies	4368814	Communicated by original authors	
TaqMan probe *PTEN*	qPCR probes	Life Technologies	Hs02621230_s1		
TaqMan probe *CNOT6L*	qPCR probes	Life Technologies	Hs00375913_m1		
TaqMan probe *VAPA*	qPCR probes	Life Technologies	Hs00427749_m1		
TaqMan probe *SERINC1*	qPCR probes	Life Technologies	Hs00380375_m1		
TaqMan control probe *ß-ACTIN*	qPCR probes	Life Technologies	Hs00969077_m1	Communicated by original authors	
TaqMan Fast Advanced Master Mix	qPCR reagent	Life Technologies	4444964	Communicated by original authors	
StepOne Plus Real-Time PCR system	Equipment	Applied Biosystems		Replaces LightCycler 480 System	
Nanodrop 2000c Spectrometer	Equipment	Thermo Scientific			
PBS	Western Reagent	Life Technologies	14190250	Communicated by original authors	
Lysis Buffer	Western Reagent	RIPA lysis buffer: 50mM Tris-HCl pH 7.4, 150mM NaCl, 1% NP-40, 0.5% sodium deoxycholate, 0.1% SDS, 5mM EDTA supplemented with protease inhibitors			
Protease inhibitors	Western Reagent	Roche Diagnostics	11873580001	Communicated by original authors	
Bradford Assay	Western Reagent	Bio-Rad		Catalog # communicated by original authors	
Bis-Tris acrylamide NuPAGE gels 4–15% Mini-PROTEAN TGX Precast Protein Gels	Western Reagent	Biorad	456–1084	Replaces NuPage gels from Life Technologies (communicated by original authors)	
Tris-Glycine SDS PAGE buffer (10x)	Western Reagent	National Diagostic	EC-870-4L	Replaces MOPS buffer from Invitrogen	
Nitrocellulose membranes	Western Reagent	Thermo Fisher Scientific	45004006	Catalog # communicated by original authors	
10xTBS buffer	Western Reagent	Biorad	170–6435	Replaces NuPage buffer from Invitrogen	
Methanol	Reagent	Pharmco	339000ACSCSGL	Communicated by original authors	
Mouse anti-HSP90 monoclonal antibody (90kDa)	Antibody	Becton Dickinson	61041	Catalog # communicated by original authors	
Rabbit anti-PTEN monoclonal antibody (54kDa)	Antibody	Cell Signaling	9559	Catalog # communicated by original authors	
Anti-mouse HRP-conjugated secondary antibody	Antibody	Abcam	Ab6728	Original not specified	
Amersham ECL Western Blotting Detection Kit	Western Blot Reagent	Amersham	RPN 2108	Replaces ECL from Applied Biological Materials	
X-ray Film (Hyblot CL, 8x10 inch)	Western Blot Reagent	Denville	E3018	Original not specified	
Spectrophotometer	Equipment	Beckman Coulter	Spectra max M2	Replaces Beckman Model DU-800 (communicated by original authors)	

#### Procedure

Notes

- All cells will be sent for mycoplasma testing and STR profiling.
- HCT116 cells (wild-type and mutant) are maintained in DMEM with 10% FBS, ^#^100 U/ml penicillin/100 µg/ml streptomycin, and ^#^2 mM glutamine at 37°C/5% CO_2_ in a humidified atmosphere.

1. Transfect HCT116 cells with siRNAs:
  - Separate HCT116 WT and DICER ^Ex5^ cells into four different cultures each.
    - These will be biological replicates.
  - For each cell type (WT and DICER Ex5) seed cells at 1.3 x 10^5^ cells per well in 12 well dishes
    - Seed 11 wells per replicate: 5 transfections x 2 wells each (one for Step 2, one for Step 3) and 1 transfection (siGlo) x 1 well.
    - Note: During the last replicate, only seed 6 wells per cell type (5 transfection conditions for Step 2) and 1 transfection condition for siGlo RISC free siRNA transfection control.
  - Transfect cells with 100 nM siRNA (or siGLO controls) using Dharmafect 1 according to manufacturer’s instructions.
    - Note: make up a separate transfection mixture for each replicate.
    - Transfect a pair of wells per replicate with each of the following:
      1. siNC
      2. siSER
      3. siVAPA
      4. siCNO
      5. siPTEN
    - Transfect a single well per replicate with the following:
      1. siGLO
    - After 24-48 hr, assess number of fluorescent cells transfected with siGLO to confirm >90% transfection efficiency.
      1. If transfection is less than 90%, record efficiency, exclude replicate and omit it from the rest of the procedure. Repeat procedure until >90% efficiency is obtained.
      2. If modification to transfection is needed, record and maintain modified steps for remaining replicates.
  - Incubate for 72 hr at 37˚C in 5% CO_2_ in a humidified atmosphere.
    - Replace growth medium after 4 hr.
2. Using one of each pair of wells (except during replicate 4), confirm siRNA knock down with qRT-PCR as in Protocol 1 Step 3. Measure mRNA expression for each siRNA transfection sample with its appropriate target and *ß-ACTIN,* and test each probe separately using RNA from the NC control transfection.
  - PTEN
  - CNOT6L
  - *VAPA*
  - SERINC1
  - *β-ACTIN* [endogenous control communicated by original author]
    - Prepare 10 µl real-time PCR reaction in triplicate for each reaction consisting of:
      1. 5 µl TaqMan mastermix
      2. 0.5 µl TaqMan probe for the gene of interest
      3. 4.5 µl cDNA (diluted 10x)
      4. Use standard TaqMan cycling protocol:
        - 50˚C 2 min
        - 95˚C 20 s
        - 40 cycles of 95˚C 1 s, 60˚C 20 s
    - Normalize each mRNA expression to *ß-ACTIN* and then normalize each siRNA to siNC for that transcript.
3. Using the second well of each pair of wells, assess PTEN protein expression by Western Blot:
  - Wash cells in chilled PBS
  - Lyse cells directly in wells by incubating on ice for 20 min with RIPA lysis buffer containing protease inhibitors.
  - Clear lysates by centrifugation at 4°C for 15 min at 12,100*xg*.
  - Determine protein concentrations with Bradford assay following manufacturer’s instructions.
  - Separate 5 µg of total protein by SDS-PAGE on 4–15% 4-15% Mini-PROTEAN TGX precast protein gels in Tris-Glycine SDS PAGE buffer.
    - HCT116 cells express high levels of PTEN protein so 5 µg should be sufficient for detection.
  - Transfer to nitrocellulose membranes in transfer buffer containing 10% methanol for 1 hr at 40V at room temperature.
    *Confirm protein transfer by Ponceau staining.
  - Block membrane with 5% milk in ^#^TBST for 30 min.
  - Probe membranes specific primary antibodies:
    - PTEN: 1:1000
    - HSP90: 1:1000
  - Wash membrane 3 times in 1X TBST for 5 min each on shaker.
  - Incubate with ^#^anti-rabbit (with PTEN primary) or ^#^anti-mouse (for HSP90 primary) HRP conjugated secondary antibody (1:2000) for 1 hr on shaker at room temperature.
  - Remove membrane from secondary antibody and wash three times in 1X TBST for 5 min each.
  - Prepare ECL solution and incubate membrane.
  - Expose membrane to X-ray film, develop and scan. Take a range of exposures (1 s, 15 s, 60 s) for each film.
    - Note from the original author: Care should be taken not to overload the gel or to overexpose the film. ceRNA regulation may only result in a 50% increase or decrease in protein levels, this difference may be overlooked if the signal is saturated and not within the dynamic range of the film.
  - Normalize PTEN to HSP90 for each sample.
4. Repeat 3 additional times.

#### Deliverables

- Data to be collected:
  - QC image data confirming transfection efficiency by measuring the number of fluorescent cells transfected with siGLO
  - QC data for total RNA (A_260/280_ and A_260/230_ absorbance ratios)
  - Raw qPCR data for each sample and a graph the mean of each target gene normalized with *β-ACTIN* and normalized relative to NC control. (Compare to Supplementary Figure 3B)
  - Full scans of each western blot with ladder (Compare to Figure 3G)
  - Raw data of band analysis and normalized bands for each sample (Compare to Figure 3H)

#### Confirmatory analysis plan

- Statistical Analysis of the Replication Data:Note: At the time of analysis, we will test for normality and homoscedasticity of the data. If the data appears skewed, we will perform the appropriate transformation to proceed with the proposed statistical analysis. If this is not possible, we will perform the equivalent non-parametric Wilcoxon-Mann-Whitney test.
  - Western blot: Two-way ANOVA of normalized PTEN levels from HCT116 cells (wild type or DICER^Ex5^ cells) transfected with siRNA for SERINC1, VAPA, CNOT6L, PTEN, or control NC followed by Bonferroni-corrected planned comparisons:
    - siNC vs. each siRNA for each cell line (8 comparisons total).
  - qRT-PCR: Bonferroni corrected one-sample *t*-tests of normalized mRNA expression in HCT116 cells (wild type or DICER^Ex5^ cells) transfected with siRNA against *SERINC1, VAPA, CNOT6L*, or *PTEN* compared to a constant (siNC=1) (8 comparisons total).
- Meta-analysis of original and replication attempt effect sizes:
  - This replication attempt will perform the statistical analysis listed above, compute the effects sizes, compare them against the reported effect size in the original paper and use a meta-analytic approach to combine the original and replication effects, which will be presented as a forest plot.

#### Known differences from the original study

All known differences are listed in the materials and reagents section above with the originally used item listed in the comments section. All differences have the same capabilities as the original and are not expected to alter the experimental design.

#### Provisions for quality control

Extracted RNA integrity will be reported with A_260/280_ and A_260/230_ absorbance ratios, and transfection efficiency will be checked using the siGLO control. The cells will be sent for mycoplasma testing confirming lack of contamination and STR profiling confirming cell line authenticity. Transfection efficiency will be recorded for each replicate and any transfection that does not contain >90% efficiency will be excluded and not continue through the rest of the procedure. If the efficiency does not reach >90%, then any modifications to the transfection protocol will be recorded. qRT-PCR will be performed to confirm silencing of mRNA expression. Images of Ponceau staining to confirm protein transfer. All data obtained from the experiment - raw data, data analysis, control data, and quality control data - will be made publicly available, either in the published manuscript or as an open access dataset available on the Open Science Framework (https://osf.io/oblj1/).

### Protocol 4: Effect of knock-down of ceRNA network genes on cell proliferation

This experiment tests the effects of siRNA-mediated depletion of *PTEN, CNOT6L*, and *VAPA* expression on cell proliferation in DU145, HCT116 WT, and HCT116 DICER^Ex5^ cells. It replicates Figure 5B.

#### Sampling

- This experiment will be repeated five (DU145 cells) times and four (HCT116 cells) times for a minimum power of 80%.
  - See Power Calculations section for details.
- Each experiment consists of DU145, HCT116 WT, and HCT116 DICER^Ex5^ cells transfected with siRNA against PTEN ceRNAs:
  - Cohort 1: siGLO RISC-free siRNA (siGLO)
  - Cohort 2: siRNA against nontargeting control 2 (siNC)
  - Cohort 3: siGENOME siRNA against *VAPA* (siVAPA)
  - Cohort 4: siGENOME siRNA against *CNOT6L* (siCNO)
  - Cohort 5: siGENOME siRNA against *PTEN* (siPTEN)
- Effects of silencing ceRNAs will be tested with
  - qRT-PCR to confirm target genes were silenced [additional QC]
  - siGLO fluorescence cell counts to confirm transfection efficiency
  - Assessment of cell proliferation (Figure 5B)

#### Materials and reagents

**Table:** 

Reagent	Type	Manufacturer	Catalog #	Comments
DU145 human prostate cancer cells	Cells	ATCC	HTB-81	
HCT116 WT and DICER^Ex5^ cells	Cells	Horizon Discovery	HD R02-019	
siGLO RISC-free siRNA siGLO	siRNA	Dharmacon	D-001600-01-05	
siGenome siRNA for nontargeting control 2	siRNA	Dharmacon	D-001210-02-05	Catalog # communicated by original authors
siGenome siRNA for *VAPA*	siRNA	Dharmacon	M-021382-01-0005	Catalog # communicated by original authors
siGenome siRNA for *CNOT6L*	siRNA	Dharmacon	M-016411-01-0005	Catalog # communicated by original authors
siGenome siRNA for *PTEN*	siRNA	Dharmacon	M-003023-02-0005	Catalog # communicated by original authors
Dulbecco's Modified Eagle's Medium (DMEM)	Cell Culture Reagent	Invitrogen	10313-039	Catalog # communicated by original authors
Fetal Bovine Serum (FBS)	Cell Culture Reagent	Invitrogen	10438-026	Catalog # communicated by original authors
Penicillin/Streptomycin	Cell Culture Reagent	Life Technologies	15140-163	Communicated by original authors
Glutamine	Cell Culture Reagent	Life Technologies	25030-081	Communicated by original authors
Trypsin	Transfection Reagent	Life Technologies	15400-054	Communicated by original authors
Dharmafect 1	Transfection Reagent	Thermo Fisher Scientific	T200104	Communicated by original authors
TRIzol reagent	qPCR reagent	Life Technologies	15596026	Communicated by original authors
RNeasy kit	qPCR reagent	Qiagen	74104	Communicated by original authors
High Capacity cDNA Archive kit	qPCR reagent	Life Technologies	4368814	Communicated by original authors
TaqMan probe *PTEN*	qPCR probes	Life Technologies	Hs02621230_s1	
TaqMan probe *CNOT6L*	qPCR probes	Life Technologies	Hs00375913_m1	
TaqMan probe *VAPA*	qPCR probes	Life Technologies	Hs00427749_m1	
TaqMan control probe *ß-ACTIN*	qPCR probes	Life Technologies	Hs00969077_m1	Additional control
TaqMan Fast Advanced Master Mix	qPCR reagent	Life Technologies	4444964	Communicated by original authors
StepOne Plus Real-Time PCR system	Equipment	Applied Biosystems		Replaces LightCycler 480 System
Nanodrop 2000C Spectrometer	Equipment	Thermo Scientific		
PBS	Western Reagent	Life Technologies	14190250	Communicated by original authors
Formalin	Fixative	Sigma Aldrich	HT501128-4l	Communicated by original authors
Crystal Violet	Stain	Sigma Aldrich	C-3886	Communicated by original authors
10% acetic acid	Solubilization reagent	Thermo Fisher Scientific	A38212	Communicated by original authors
BioTek Synergy HT Multi-mode Microplate Reader	Equipment	BioTek Instrument		Replaces Beckman Coulter Model DU-800 (communicated by original authors)

#### Procedure

Notes:

- HCT116 cells (wild-type and mutant) are maintained in DMEM with 10% FBS, ^#^100 U/ml penicillin/100 µg/ml streptomycin, and ^#^2 mM glutamine at 37°C/5% CO_2_ in a humidified atmosphere.
- DU145 cells are maintained in DMEM supplemented with 10% FBS, ^#^100 U/ml penicillin/100 µg/ml streptomycin, and ^#^2 mM glutamine at 37˚C in 5% CO_2_ in a humidified atmosphere.
- All cells will be sent for mycoplasma testing and STR profiling.

1. Transfect DU145, HCT116 WT, and HCT116 DICER^Ex5^ cells with siRNAs
  - Separate DU145 into five cultures each, and HCT116 WT, and HCT116 DICER^Ex5^ cells each into 4 different cultures.
    - These will be biological replicates for each cell line.
  - Seed cells 1.3 x 10^5^ cells per well of a 12-well plate for subsequent experiments:
    - For measuring transfection efficiency (Step 1c ii):
      1. Seed 1 well (Cohort 1) per replicate per cell line.
        - 5 wells for DU145 cells
        - 4 wells for HCT116 WT cells
        - 4 wells for HCT116 Dicer^Ex5^ cells
    - For cell proliferation assay (Step 2).
      1. Seed 4 wells (Cohorts 2-5) per replicate per cell line.
        - 20 wells for DU145 cells
        - 16 wells for HCT116 WT cells
        - 16 wells for HCT116 Dicer^Ex5^ cells
    - For qPCR confirmation of siRNA knockdown (Step 3).
      1. Seed 4 wells (Cohorts 2-5) per replicate per cell line.
        - 20 wells for DU145 cells
        - 16 wells for HCT116 WT cells
        - 16 wells for HCT116 Dicer^Ex5^ cells
  - Transfect wells with 100 nM of appropriate siRNA using Dharmafect1 according to manufacturer’s instructions.
    - Note: make up a separate transfection mix for each biological replicate.
    - Incubate wells for measuring transfection efficiency for 24-48 hr, then assess number of fluorescent cells transfected with siGLO to confirm >90% transfection efficiency.
      1. If transfection is less than 90%, record efficiency for attempt, exclude attempt and do not continue with the rest of the procedure. Repeat procedure until >90% efficiency is obtained.
      2. If modification to transfection is needed during first attempt(s), record and maintain modified steps for remaining replicates.
    - Incubate wells for seeding the cell proliferation assay for 8 hr, then proceed to Step 2.
    - Incubate wells for qPCR for 72 hr, then proceed to Step 3.
2. Measure cell proliferation
  - Eight hours after transfection, trypsinize and resuspend cells. Split each well into 1 well each of four 12-well plates, seeding 20,000 cells/well. Incubate overnight.
    - Two 12-well plates (a set) will provide sufficient wells to accommodate all replicates for one day of the time course per cell line.
    - 8 plates will be needed per cell line for a full 4 day time course.
  - Starting on the following day (d0), fix one set of plates per cell line per day.
    - Wash cells with PBS.
    - Fix cells in 10% formalin solution for 10 min at room temperature.
    - Store cells in PBS at 4°C until all plates are fixed.
      1. Plates should be collected on day 0, 1, 2 and 3.
  - c. On day 3, stain all wells of all plates with crystal violet.
    - Add 1 ml 0.1% Crystal Violet solution in 20% methanol.
    - Shake gently for 15 min at room temperature.
    - Wash 2 times in distilled water and let plates dry completely.
    - Solubilize remaining crystal violet by adding 1 ml of 10% acetic acid to each well.
    - Shake gently for 15 min at room temperature.
    - Transfer 100 µl to a 96-well plate and measure OD at 595 nm in a plate reader.
3. Confirm siRNA knock down with qPCR as in Protocol 1 Step 3. Perform qRT-PCR to measure mRNA expression for each siRNA transfection sample with its appropriate target and *ß-ACTIN*, and test each probe separately using RNA from the NC control transfection.
  - PTEN
  - *CNOT6L*
  - *VAPA*
  - *ß-ACTIN* [endogenous control communicated by original author]
    1. Prepare 10 µl real-time PCR reaction in triplicate for each reaction consisting of:
      - 5 µl TaqMan mastermix
      - 0.5 µl TaqMan probe for the gene of interest
      - 4.5 µl cDNA (diluted 10x)
      - Use standard TaqMan cycling protocol:
        - 50˚C 2 min
        - 95˚C 20 s
        - 40 cycles of 95˚C 1 s, 60˚C 20 s

#### Deliverables

- Data to be collected:
  - QC image data confirming transfection efficiency by measuring the number of fluorescent cells transfected with siGLO
  - QC data for total RNA (A_260/280_ and A_260/230_ absorbance ratios)
  - Raw qPCR data for each sample and a graph of the mean of each target gene normalized with *ß-ACTIN* and graphed relative to NC control.
  - Raw numbers for optical density measures of colonies for each sample.

#### Confirmatory analysis plan

- Statistical Analysis of the Replication Data:Note: At the time of analysis, we will test for normality and homoscedasticity of the data. If the data appears skewed, we will perform the appropriate transformation in order to proceed with the proposed statistical analysis. If this is not possible we will perform the equivalent non-parametric Wilcoxon-Mann-Whitney test.
  - Cell proliferation data: One-way ANOVA of AUC values of DU145 cells transfected with siRNA for *VAPA, CNOT6L, PTEN*, or siNC followed by Bonferroni-corrected planned comparisons:
    - siNC vs each siRNA (3 comparisons total).
  - Cell proliferation data: Two-way ANOVA of AUC values of HCT116^WT^ or HCT116 DICER^Ex5^ cells transfected with siRNA for *VAPA, CNOT6L, PTEN*, or siNC followed by Bonferroni-corrected planned comparisons:
    - siNC vs. each siRNA, for each cell line (6 comparisons total).
- Meta-analysis of original and replication attempt effect sizes:
  - This replication attempt will perform the statistical analysis listed above, compute the effects sizes, compare them against the reported effect size in the original paper and use a meta-analytic approach to combine the original and replication effects, which will be presented as a forest plot.
- Additional exploratory analysis:siRNA knockdown confirmation [additional control]
  - Two-way ANOVA of mRNA expression in HCT116 cells (wild type or DICER^Ex5^ cells) transfected with siRNA against NC, *VAPA, CNOT6L*, or *PTEN*, with the following Bonferroni-corrected comparisons:
    - Non-coding siRNA vs. each of the ceRNA transfected cells (3 comparisons total).

#### Known differences from the original study

All known differences are listed in the materials and reagents section above, with the originally used item listed in the comments section. All differences have the same capabilities as the original and are not expected to alter the experimental design.

#### Provisions for quality control

Extracted RNA integrity will be reported with A_260/280_ and A_260/230_ absorbance ratios, and transfection efficiency will be checked using the siGLO control. Cells will be sent for mycoplasma testing confirming lack of contamination and STR profiling confirming cell line authenticity. Transfection efficiency will be recorded for each replicate and any transfection that does not contain >90% efficiency will be excluded and not continue through the rest of the procedure. Any modifications to the transfection protocol will be recorded and the procedure will be maintained for the remaining replicates. All data obtained from the experiment - raw data, data analysis, control data and quality control data - will be made publicly available, either in the published manuscript or as an open access dataset available on the Open Science Framework (https://osf.io/oblj1/).

### Protocol 5: Knock-down of ceRNA network genes results in AKT activation

This experiment tests the effects of siRNA-mediated depletion of *PTEN, CNOT6L*, and *VAPA* expression on AKT activation in DU145, HCT116 WT, and HCT116 Dicer^Ex5^ cells. It replicates Figure 5A.

#### Sampling

- This experiment will be repeated at least 7 times for a minimum power of 80%. The original Western blot data is qualitative, thus to determine an appropriate number of replicates to initially perform, sample sizes based on a range of potential variance was determined.
  - See Power Calculations section for details.
- Each experiment consists of DU145, HCT116 WT, and HCT116 DICER^Ex5^ cells transfected with siRNA against *PTEN* ceRNAs:
  - Cohort 1: siGLO RISC-free siRNA (siGLO)
  - Cohort 2: siRNA against nontargeting control 2 (siNC)
  - Cohort 3: siGENOME siRNA against *VAPA* (siVAPA)
  - Cohort 4: siGENOME siRNA against *CNOT6L* (siCNO)
  - Cohort 5: siGENOME siRNA against *PTEN* (siPTEN)
- Effects of silencing ceRNAs will be tested with
  - qRT-PCR to confirm target genes were silenced [additional QC]
  - siGLO fluorescence cell counts to confirm transfection efficiency
  - Assessment of AKT phosphorylation by Western blot (Figure 5A)

#### Materials and reagents

**Table:** 

Reagent	Type	Manufacturer	Catalog #	Comments
DU145 human prostate cancer cells	Cells	ATCC	HTB-81	
HCT116 WT and DICER^Ex5^ cells	Cells	Horizon Discovery	HD R02-019	
siGLO RISC-free siRNA siGLO	siRNA	Dharmacon	D-001600-01-05	
siGenome siRNA for nontargeting control 2	siRNA	Dharmacon	D-001210-02-05	Catalog # communicated by original authors
siGenome siRNA for *VAPA*	siRNA	Dharmacon	M-021382-01-0005	Catalog # communicated by original authors
siGenome siRNA for *CNOT6L*	siRNA	Dharmacon	M-016411-01-0005	Catalog # communicated by original authors
siGenome siRNA for *PTEN*	siRNA	Dharmacon	M-003023-02-0005	Catalog # communicated by original authors
TaqMan probe *PTEN*	qPCR probes	Life Technologies	Hs02621230_s1	
TaqMan probe *CNOT6L*	qPCR probes	Life Technologies	Hs00375913_m1	
TaqMan probe *VAPA*	qPCR probes	Life Technologies	Hs00427749_m1	
TaqMan control probe *ß-ACTIN*	qPCR probes	Life Technologies	Hs00969077_m1	Additional control
TaqMan Fast Advanced Master Mix	qPCR reagent	Life Technologies	4444964	Communicated by original authors
StepOne Plus Real-Time PCR system	Equipment	Applied Biosystems		Replaces LightCycler 480 System
Nanodrop 2000C Spectrometer	Equipment	Thermo Scientific		
Dulbecco's Modified Eagle's Medium (DMEM)	Cell Culture Reagent	Invitrogen	10313-039	Catalog # communicated by original authors
Fetal Bovine Serum (FBS)	Cell Culture Reagent	Invitrogen	10438-026	Catalog # communicated by original authors
Penicillin/Streptomycin	Cell Culture Reagent	Life Technologies	15140-163	Communicated by original authors
Glutamine	Cell Culture Reagent	Life Technologies	25030-081	Communicated by original authors
Trypsin	Transfection Reagent	Life Technologies	15400-054	Communicated by original authors
Dharmafect 1	Transfection Reagent	Thermo Fisher Scientific	T200104	Communicated by original authors
PBS	Western Reagent	Life Technologies	14190250	Communicated by original authors
Lysis Buffer	Western Reagent	RIPA lysis buffer: 50mM Tris-HCl pH 7.4, 150mM NaCl, 1% NP-40, 0.5% sodium deoxycholate, 0.1% SDS, 5mM EDTA supplemented with proteinase inhibitors		
Protease inhibitors	Western Reagent	Roche Diagnostics	11873580001	Communicated by original authors
Bradford Dye	Western Reagent	Bio-Rad	500-0006	Catalog # communicated by original authors
4–15% Mini-PROTEAN TGX Precast Protein Gels	Western Reagent	Biorad	456–1084	Replaces NuPage gels from Life Technologies (communicated by original authors)
Tris-Glycine SDS PAGE buffer (10x)	Western Reagent	National Diagnostic	EC-870-4L	Replaces MOPS buffer from Invitrogen
Nitrocellulose membranes	Western Reagent	Thermo Fisher Scientific	45004006	Catalog # communicated by original authors
10xTBS buffer	Western Reagent	Biorad	170–6435	Replaces NuPage buffer from Invitrogen
Methanol	Chemical	Pharmco	339000ACSCSGL	Communicated by original authors
Rabbit anti-pAKT (Ser473) polyclonal antibody (60kDa)	Antibody	Cell Signaling	9271	Catalog # communicated by original authors
Rabbit anti-AKT polyclonal antibody (60kDa)	Antibody	Cell Signaling	9272	Catalog # communicated by original authors
Amersham ECL Western Blotting Detection Kit	Western Blot Reagent	Amersham	RPN2108	Replaces ECL from Applied Biological Materials
Spectrophotometer	Equipment	Beckman Coulter	Spectra max M2	Replaces Beckman Model DU-800 (communicated by original authors)

#### Procedure

##### Notes:

- HCT116 cells (wild-type and mutant) are maintained in DMEM with 10% FBS, ^#^100 U/ml penicillin/100 µg/ml streptomycin, and ^#^2 mM glutamine at 37°C/5% CO_2_ in a humidified atmosphere.
- DU145 cells are maintained in DMEM supplemented with 10% FBS, ^#^100 U/ml penicillin/100 µg/ml streptomycin, and ^#^2 mM glutamine at 37˚C in 5% CO_2_ in a humidified atmosphere.
- All cells will be sent for mycoplasma testing and STR profiling.

1. Transfect DU145, HCT116 WT, and HCT116 DICER^Ex5^ cells with siRNAs
  - Seed cells for subsequent experiments with 1.3 x 10^5^ cells per well in a 12-well plate:
    - For measuring transfection efficiency (Step 1c ii):
      1. Seed 1 well (Cohort 1) per replicate.
        - DU145 cells
        - HCT116 WT cells
        - HCT116 Dicer^Ex5^ cells
    - For AKT activation and Western blot (Step 2).
      1. Seed 3 well (Cohort 2-5) per replicate.
        - 12 wells for DU145 cells
        - 12 wells for HCT116 WT cells
        - 12 wells for HCT116 Dicer^Ex5^ cells
  - Transfect wells with 100 nM of appropriate siRNA using Dharmafect1 according to manufacturer’s instructions.
    - Note: make up a separate transfection mix for each biological replicate.
    - Incubate wells for measuring transfection efficiency for 24-48 hr, then assess number of fluorescent cells transfected with siGLO to confirm >90% transfection efficiency.
      1. If transfection is less than 90%, record efficiency, exclude attempt and do not continue with the rest of the procedure. Repeat procedure until >90% efficiency is obtained.
      2. If modification to transfection is needed, record and maintain modified steps for remaining replicates.
2. Stimulate activation of AKT then measure levels of phosphorylated AKT by Western blot.
  - After 72 hr, serum-starve cells overnight: replace media with serum-free media and incubate overnight (approximately 16 hr).
  - The following morning, harvest one well at 0 min (pre-stimulation), re-stimulate the remaining cells by adding the appropriate volume of warmed 100% FBS to existing media in each trio of matched wells for a 10% final concentration. Incubate wells for 5 or 15 min.
    - Harvest one well at 5 min and one well at 15 min post FBS addition.
  - Harvest cells and perform Western blot as specified in Protocol 3 step 3.
    - Note: load 10 µg of protein per well.
    - Probe membranes specific primary antibodies
      1. pAKT (Ser473); 1:1000
      2. total AKT; 1:1000
        - Loading control
    - Note from original author: Phosphorylated proteins are less stable in lysis buffer than non-phosphorylated proteins. Try to use fresh lysates for subsequent western blotting as far as possible. Transfer samples to the protein loading buffer as fast as possible and keep freeze thaw cycles to an absolute minimum.
  - Normalize pAKT to total AKT for each sample.
3. Repeat at least 6 additional times.

#### Deliverables

- Data to be collected:
  - QC image data confirming transfection efficiency by measuring the number of fluorescent cells transfected with siGLO
  - QC data for total RNA (A_260/280_ and A_260/230_ absorbance ratios)
  - Raw qPCR data for each sample and a graph of the mean of each target gene normalized with *ß-ACTIN* and graphed relative to NC control.
  - Full scans of all films for each western including ladder.

#### Confirmatory analysis plan

- Statistical Analysis of the Replication Data:Note: At the time of analysis, we will test for normality and homoscedasticity of the data. If the data appears skewed, we will perform the appropriate transformation to proceed with the proposed statistical analysis. If this is not possible, we will perform the equivalent non-parametric Wilcoxon-Mann-Whitney test.
  - Two-way ANOVA of normalized pAKT levels of DU145 cells transfected with siRNA for *VAPA, CNOT6L, PTEN*, or siNC measured at 0 min, 5 min, and 15 min followed by Bonferroni-corrected planned contrasts:
    - siNC vs each siRNA, collapsed across all times (3 contrasts total).
  - Three-way ANOVA (3x4x2) of normalized pAKT levels of HCT116^WT^ or HCT116 DICER^Ex5^ cells transfected with siRNA for *VAPA, CNOT6L, PTEN*, or siNC measured at 0 min, 5 min, and 15 min:
    - HCT116^WT^ cells with the following Bonferroni-corrected planned contrasts:
      - siNC vs. each siRNA, collapsed across all times (3 contrasts total).
    - HCT116 DICER^Ex5^ cells with the following Bonferroni-corrected planned contrasts:
      - siNC vs. each siRNA, collapsed across all times (3 contrasts total).
- Meta-analysis of original and replication attempt effect sizes:
  - The replication data (mean and 95% confidence interval) will be plotted with the original reported data value plotted as a single point on the same plot for comparison.

#### Known differences from the original study

All known differences are listed in the materials and reagents section above, with the originally used item listed in the comments section. All differences have the same capabilities as the original and are not expected to alter the experimental design.

#### Provisions for quality control

The cells will be sent for mycoplasma testing confirming lack of contamination and STR profiling confirming cell line authenticity. Transfection efficiency will be recorded for each replicate and any transfection that does not contain >90% efficiency will be excluded and not continue through the rest of the procedure. Any modifications to the transfection protocol will be recorded, and the procedure will be maintained for the remaining replicates. Images of Ponceau staining to confirm protein transfer. All data obtained from the experiment - raw data, data analysis, control data, and quality control data - will be made publicly available, either in the published manuscript or as an open access dataset available on the Open Science Framework (https://osf.io/oblj1/).

#### Power calculations

For additional details on power calculations, please see analysis scripts and associated files on the Open Science Framework:

https://osf.io/c8hb5

### Protocol 1

Summary of original luciferase activity data:

- Note: data provided by original authors for Figure 3C

**Table:** 

siRNA	Luciferase activity	SD	N
siNC	100	9.28	4
siSER	70.29	6.99	4
siZNF	108.62	9.2	4
siVAPA	47.54	2.89	4
siCNO	69.82	3.69	4
siPTEN	20.32	1.11	4

#### Test family

- 2 tailed *t* test, Wilcoxon-Mann-Whitney test, Bonferroni’s correction: alpha error = 0.01

Power Calculations performed with G*Power software, version 3.1.7 (Faul et al., 2007).

**Table:** 

Group 1	Group 2	Effect size *d*	A priori power	Group 1 sample size	Group 2 sample size
siNC	siSER	3.61647	81.9%	4	4
siNC	siZNF	0.9329	80.2%^2^	30^2^	30^2^
siNC	siVAPA	7.63300	98.5%^1^	3^1^	3^1^
siNC	siCNO	4.27377	93.2%	4	4
siNC	siPTEN	12.0568	99.9%^1^	3^1^	3^1^

^1^ 4 samples per group will be used making the power >99.9%.

^2^ A sensitivity calculation was performed since the original data showed a non-significant effect. The effect size that can be detected with 80% power and a sample size n=4 per group is 3.5378.

#### Test family

- Due to the large variance, the following parametric tests are only used for comparison purposes. The sample size is based on the non-parametric tests listed above.
- ANOVA: Fixed effects, omnibus, one-way: alpha error = 0.05

#### Power calculations

- Performed with G*Power software, version 3.1.7 (Faul et al., 2007).
- ANOVA F test statistic and partial η^2 ^performed with R software, version 3.1.2 (R Core Team 2015).

**Table:** 

Groups	F test statistic	Partial η^2^	Effect size *f*	A priori power	Total sample size
siRNA silencing groups	F_(5,18)=_106.0	0.9672	5.4302	>99.9%	12^1^

^1^ 24 total samples (4 per group) will be used based on the planned comparisons making the power >99.9%.

#### Test family

- Two-tailed *t* test, difference between two independent means, Bonferroni correction: alpha error = 0.01

#### Power calculations

- Performed with G*Power software, version 3.1.7 (Faul et al., 2007).

**Table:** 

Group 1	Group 2	Effect size *d*	A priori power	Group 1 sample size	Group 2 sample size
siNC	siSER	3.61647	85.8%	4	4
siNC	siZNF	0.9329	80.8%^2^	29^2^	29^2^
siNC	siVAPA	7.63300	99.4%^1^	3^1^	3^1^
siNC	siCNO	4.27377	95.4%	4	4
siNC	siPTEN	12.0568	99.9%^1^	3^1^	3^1^

^1^ 4 samples per group will be used making the power >99.9%.

^2^ A sensitivity calculation was performed since the original data showed a non-significant effect. The effect size that can be detected with 80% power and a sample size n=4 per group is 3.3711.

Summary of original qPCR gene expression data:

- Note: data provided by original authors for Figure S3A
- We estimated SD to be 0.001, when it was reported as zero.

**Table:** 

siRNA	mRNA expression	SD	Assumed N
siSER	0.03	0.001	4
siZNF	0.35	0.11	4
siVAPA	0.03	0.001	4
siCNO	0.07	0.01	4
siPTEN	0.1	0.04	4

#### Test family

- 2 tailed *t* test, Wilcoxon-Signed Ranks one-sample test, Bonferroni’s correction: alpha error = 0.01

Power Calculations performed with G*Power software, version 3.1.7 (Faul et al., 2007).

**Table:** 

Group	Effect size *d*	A priori power	Sample size
siSER	970.00	99.9%	3
siZNF	5.91	97.7%	4
siVAPA	970.00	99.9%	3
siCNO	93.00	99.9%	3
siPTEN	22.50	99.9%	3

#### Test family

- Due to the large variance, the following parametric tests are only used for comparison purposes. The sample size is based on the non-parametric tests listed above.
- Two-tailed *t* test, difference from a constant, Bonferroni correction: alpha error = 0.01

#### Power calculations

- Performed with G*Power software, version 3.1.7 (Faul et al., 2007).

**Table:** 

Group	Effect size *d*	A priori power	sample size
siSER	970.00	99.9%	3
siZNF	5.91	99.0%	4
siVAPA	970.00	99.9%	3
siCNO	93.00	99.9%	3
siPTEN	22.50	99.9%	3

### Protocol 2

Summary of original Luciferase data:

- Note: data provided by original authors for Figure 3D.

**Table:** 

siRNA	Luciferase Activity	SD	N
Empty Vector	100	8.83	4
SER 3'U	127.86	11.59	4
VAPA 3'U1	140.84	17.8	4
VAPA 3'U2	150.25	9.37	4
CNO 3'U1	142.91	9.92	4
CNO 3'U2	145.88	10.59	4
PTEN 3'U	153.32	2.06	4

#### Test family

- 2 tailed *t* test, Wilcoxon-Mann-Whitney test, Bonferroni’s correction: alpha error = 0.00833

Power Calculations performed with G*Power software, version 3.1.7 (Faul et al., 2007).

**Table:** 

Group 1	Group 2	Effect size *d*	A priori power	Group 1 sample size	Group 2 sample size
Empty Vector	SER 3'U	2.70411	85.7%	6	6
Empty Vector	VAPA 3'U1	2.90675	91.0%	6	6
Empty Vector	VAPA 3'U2	5.51955	81.3%^1^	3^1^	3^1^
Empty Vector	CNO 3'U1	4.56935	94.6%^1^	4^1^	4^1^
Empty Vector	CNO 3'U2	4.70574	95.7%^1^	4^1^	4^1^
Empty Vector	PTEN 3'U	8.31642	99.0%^1^	3^1^	3^1^

^1^ 6 samples per group will be used making the power >99.9%.

#### Test family

- Due to the large variance, the following parametric tests are only used for comparison purposes. The sample size is based on the non-parametric tests listed above.
- ANOVA: Fixed effects, omnibus, one-way: alpha error = 0.05

#### Power calculations

- Performed with G*Power software, version 3.1.7 (Faul et al., 2007).
- ANOVA F test statistic and partial η^2^performed with R software, version 3.1.2 (R Core Team 2015).

**Table:** 

Groups	F test statistic	Partial η^2^	Effect size *f*	A priori power	Total sample size
PTEN ceRNAs 3’UTRs	F_(6, 21)=_11.347	0.7643	5.4302	99.9%	14^1^

^1^ 42 total samples (6 per group) will be used based on the planned comparisons making the power >99.9%.

#### Test family

- Two-tailed *t* test, difference between two independent means, Bonferroni correction: alpha error = 0.00833

#### Power calculations

- Performed with G*Power software, version 3.1.7 (Faul et al., 2007).

**Table:** 

Group 1	Group 2	Effect size *d*	A priori power	Group 1 sample size	Group 2 sample size
Empty Vector	SER 3'U	2.70411	88.5%	6	6
Empty Vector	VAPA 3'U1	2.90675	82.4%^1^	5^1^	5^1^
Empty Vector	VAPA 3'U2	5.51955	86.9%^2^	3^2^	3^2^
Empty Vector	CNO 3'U1	4.56935	96.5%^2^	4^2^	4^2^
Empty Vector	CNO 3'U2	4.70574	97.4%^2^	4^2^	4^2^
Empty Vector	PTEN 3'U	8.31642	99.6%^2^	3^2^	3^2^

^1^ 6 samples per group will be used making the power 96.2%.

^2^ 6 samples per group will be used making the power 99.9%.

### Protocol 3

Summary of original Western blot data:

- Note: data provided by original authors for Figure 3H.

**Table:** 

siRNA	Cell type	PTEN expression	SD	N
siNC	WT	100	8.3	4
	DicerEx5	100	4.8	4
siSER	WT	52.6	8.9	4
	DicerEx5	117	6.5	4
SiVAPA	WT	51.7	6.5	4
	DicerEx5	107.5	9.4	4
siCNO	WT	58.7	4.5	4
	DicerEx5	113	4.4	4
siPTEN	WT	1.9	0.2	4
	DicerEx5	1.3	0.001	4

#### Test family

- 2 tailed *t* test, Wilcoxon-Mann-Whitney test, Bonferroni’s correction: alpha error = 0.00625

Power Calculations performed with G*Power software, version 3.1.7 (Faul et al., 2007).

**Table:** 

Group 1	Group 2	Effect size *d*	A priori power	Group 1 sample size	Group 2 sample size
WT siNC	WT siSER	5.50828	98.4%	4	4
WT siNC	WT siVAPA	6.47928	87.2%^1^	3^1^	3^1^
WT siNC	WT siCNO	6.18627	83.9%^1^	3^1^	3^1^
WT siNC	WT siPTEN	16.71013	99.9%^1^	3^1^	3^1^
Sensitivity Calculations		Detectable Effect size *d*	A priori power	Group 1 sample size	Group 2 sample size
Dicer siNC	Dicer siSER	3.895	80%	4	4
Dicer siNC	Dicer siVAPA	3.895	80%	4	4
Dicer siNC	Dicer siCNO	3.895	80%	4	4
Dicer siNC	Dicer siPTEN	3.895	80%	4	4

^1^ 4 samples per group will be used making the power >99%.

#### Test family

- Due to the large variance, the following parametric tests are only used for comparison purposes. The sample size is based on the non-parametric tests listed above.
- Two-way ANOVA: Fixed effects, main effects, special and interactions: alpha error = 0.05

#### Power calculations

- Performed with G*Power software, version 3.1.7 (Faul et al., 2007).
- ANOVA F test statistic and partial η^2^performed with R software, version 3.1.2 (Team 2014; R Core Team 2015).

**Table:** 

Groups	F test statistic	Partial η^2^	Effect size *f*	A priori power	Total sample size
siRNA silencing groups in WT or DicerEx5 cells	F_(4,30)_= 54.237 (interaction)	0.87852	2.6892	89.9%^1^	14^1^

^1^ 40 total samples (4 per group) will be used based on the planned comparisons making the power >99.9%.

#### Test family

- Two-tailed *t* test, difference between two independent means, Bonferroni correction: alpha error = 0.00625

#### Power calculations

- Performed with G*Power software, version 3.1.7 (Faul et al., 2007).

**Table:** 

Group 1	Group 2	Effect size *d*	A priori power	Group 1 sample size	Group 2 sample size
WT siNC	WT siSER	5.50828	81.1%	3^1^	3^1^
WT siNC	WT siVAPA	6.47928	92.0%	3^1^	3^1^
WT siNC	WT siCNO	6.18627	89.5%	3^1^	3^1^
WT siNC	WT siPTEN	16.71013	82.6%^1^	2^1^	2^1^
Sensitivity Calculations		Detectable Effect size *d*	A priori power	Group 1 sample size	Group 2 sample size
Dicer siNC	Dicer siSER	3.697	80%	4	4
Dicer siNC	Dicer siVAPA	3.697	80%	4	4
Dicer siNC	Dicer siCNO	3.697	80%	4	4
Dicer siNC	Dicer siPTEN	3.697	80%	4	4

^1^ 4 samples per group will be used making the power >99.9%.

Summary of original mRNA expression data:

- Note: data provided by original authors for Figure S3B.

**Table:** 

siRNA	Cell Type	mRNA expression	SD	N
siSER	WT	0.036	0.0049	4
	DicerEx5	0.028	0.0007	4
SiVAPA	WT	0.027	0.0019	4
	DicerEx5	0.034	0.0005	4
siCNO	WT	0.107	0.033	4
	DicerEx5	0.033	0.0025	4
siPTEN	WT	0.075	0.0237	4
	DicerEx5	0.115	0.0414	4

#### Test family

- 2 tailed *t* test, Wilcoxon-Signed Ranks one-sample test, Bonferroni’s correction: alpha error = 0.00625

#### Power calculations

- Performed with G*Power software, version 3.1.7 (Faul et al., 2007).

**Table:** 

Group	Effect size *d*	A priori power	Group 1 sample size
WT siSER	196.75	99.5%	3
WT siVAPA	512.00	99.5%	3
WT siCNO	27.06	99.5%	3
WT siPTEN	39.03	99.5%	3
Dicer siSER	1388.50	99.5%	3
Dicer siVAPA	1932.00	99.5%	3
Dicer siCNO	386.80	99.5%	3
Dicer siPTEN	21.38	99.5%	3

#### Test family

- Due to the large variance, the following parametric tests are only used for comparison purposes. The sample size is based on the non-parametric tests listed above.
- Two-tailed *t* test, difference from a constant (mu=1), Bonferroni correction: alpha error = 0.00625

#### Power calculations

- Performed with G*Power software, version 3.1.7 (Faul et al., 2007).

**Table:** 

Group	Effect size *d*	A priori power	Group 1 sample size
WT siSER	196.75	99.9%	3
WT siVAPA	512.00	99.9%	3
WT siCNO	27.06	99.9%	3
WT siPTEN	39.03	99.9%	3
Dicer siSER	1388.50	99.9%	3
Dicer siVAPA	1932.00	99.9%	3
Dicer siCNO	386.80	99.9%	3
Dicer siPTEN	21.38	99.9%	3

### Protocol 4

Summary of original cell proliferation data:

- Note: data of mean values provided by original authors for Figure 5B.

**Table:** 

		Cell Proliferation (Optical Density)			
Cell Type	siRNA	Day 0	Day 1	Day 2	Day 3
DU145	siNC	0	0.08	0.37	0.92
	siPTEN	0	0.16	0.83	1.96
	siCNO	0	0.06	0.63	1.66
	siVAPA	0	0.12	0.78	1.75
HCT116 WT	siNC	0	0.30	0.91	1.35
	siPTEN	0	0.60	1.63	2.07
	siCNO	0	0.77	1.98	2.19
	siVAPA	0	0.66	1.65	1.98
HCT116 Dicer Ex5	siNC	0	0.12	0.49	0.74
	siPTEN	0	0.69	1.72	1.90
	siCNO	0	0.49	1.09	1.75
	siVAPA	0	0.30	0.95	1.34

- Area under the curve calculation with R software, version 3.1.2 (R Core Team 2015).

**Table:** 

Cell Type	siRNA	Area under the curve	SD	N
DU145	siNC	0.910	0.235	3
	siPTEN	1.970	0.140	3
	siCNO	1.520	0.141	3
	siVAPA	1.775	0.076	3
HCT116 WT	siNC	1.885	0.180	3
	siPTEN	3.265	0.156	3
	siCNO	3.845	0.290	3
	siVAPA	3.300	0.275	3
HCT116 Dicer Ex5	siNC	0.980	0.012	3
	siPTEN	3.360	0.310	3
	siCNO	2.455	0.145	3
	siVAPA	1.920	0.285	3

### DU145 cells

#### Test family

- 2 tailed, Wilcoxon-Mann-Whitney test, Bonferroni’s correction: alpha error = 0.0167

Power Calculations performed with G*Power software, version 3.1.7 (Faul et al., 2007).

**Table:** 

Group 1	Group 2	Effect size *d*	A priori power	Group 1 sample size	Group 2 sample size
siNC	siPTEN	5.115	89.5%	3	3
siNC	siCNO	2.944	89.4%	5	5
siNC	siVAPA	4.174	96.3%	4	4

#### Test family

- Due to the large variance, the following parametric tests are only used for comparison purposes. The sample size is based on the non-parametric tests listed above.
- One way ANOVA: Fixed effects, special, main effects and interactions, alpha error = 0.05

#### Power calculations

- Performed with G*Power software, version 3.1.7 (Faul et al., 2007).
- ANOVA F test statistic and partial η^2 ^performed with R software, version 3.1.2 (R Core Team 2015).

**Table:** 

Groups	F test statistic	Partial η2	Effect size f	A priori power	Total sample size
Optical density of DU145 cells transfected with siRNAs	F(6, 24) =14.26	0.7810	1.8884	82.73%	16^1^

^1^60 total samples (5 per group) will be used based on the planned comparisons making the power >99.99%.

#### Test family

- Two-tailed *t* test, difference between two independent means, Bonferroni correction: alpha error = 0.0167

#### Power calculations

- Performed with G*Power software, version 3.1.7 (Faul et al., 2007).

**Table:** 

Cells	Group 1	Group 2	Effect size *d*	A priori power	Group 1 sample size	Group 2 sample size
DU145	siNC	siPTEN	5.115	92.9%^1^	3^1^	3^1^
	siNC	siCNO	2.944	91.7%	5	5
	siNC	siVAPA	4.174	97.6%^1^	4^1^	4^1^

^1^5 samples per group will be used making the power >99%.

### HCT116 cells

#### Test family

- 2 tailed, Wilcoxon-Mann-Whitney test, Bonferroni’s correction: alpha error = 0.00833

Power Calculations performed with G*Power software, version 3.1.7 (Faul et al., 2007).

**Table:** 

Cells	Group 1	Group 2	Effect size *d*	A priori power	Group 1 sample size	Group 2 sample size
HCT116 WT	siNC	siPTEN	6.695	93.4%	3	3
	siNC	siCNO	3.853	84.1%	4	4
	siNC	siVAPA	5.463	80.5%	3	3
HCT116 Dicer Ex5	siNC	siPTEN	10.452	99.9%	3	3
	siNC	siCNO	6.478	91.8%	3	3
	siNC	siVAPA	4.128	89.1%	4	4

#### Test family

- Due to the large variance, the following parametric tests are only used for comparison purposes. The sample size is based on the non-parametric tests listed above.
- Two way ANOVA: Fixed effects, special, main effects and interactions, alpha error = 0.05

#### Power calculations

- Performed with G*Power software, version 3.1.7 (Faul et al., 2007).
- ANOVA F test statistic and partial η^2^performed with R software, version 3.1.2 (R Core Team 2015).

**Table:** 

Groups	F test statistic	Partial η2	Effect size f	A priori power	Total sample size
Optical density of HCT116 WT, and DICEREx5 cells transfected with siRNAs	F(3, 23) =14.08	0.7253	1.6249	81.69%	12^1^

^1^24 total samples (3 per group) will be used based on the planned comparisons making the power >99.99%.

#### Test family

- Two-tailed *t* test, difference between two independent means, Bonferroni correction: alpha error = 0.00833

#### Power calculations

- Performed with G*Power software, version 3.1.7 (Faul et al., 2007).

**Table:** 

Cells	Group 1	Group 2	Effect size *d*	A priori power	Group 1 sample size	Group 2 sample size
HCT116 WT	siNC	siPTEN	6.695	96.3%	3	3
	siNC	siCNO	3.853	87.9%	3	3
	siNC	siVAPA	5.463	86.2%	3	3
Sensitivity Calculations			Detectable Effect size *d*	A priori power	Group 1 sample size	Group 2 sample size
HCT116 Dicer Ex5	siNC	siPTEN	3.495	80%	4	4
	siNC	siCNO	3.495	80%	4	4
	siNC	siVAPA	3.495	80%	4	4

### Protocol 5

Summary of original AKT Activation data

- Note: data provided by original authors for Figure 5A.
  - We used the average band intensity for siNC since they were measured twice.

**Table:** 

		pAkt/Total Akt		
Cell Type	siRNA	0 min	5 min	15 min
DU145	siNC	1	3.2	1.95
	siPTEN	5	8.1	6.9
	siCNO	0.8	4.1	3.4
	siVAPA	2.1	10.9	6.6
HCT116 WT	siNC	1	2.35	2.45
	siPTEN	6.3	9.5	9.5
	siCNO	1.2	4.1	3
	siVAPA	1.8	4.4	3.5
HCT116 Dicer Ex5	siNC	1	5.15	2.25
	siPTEN	5.3	14.7	7.2
	siCNO	0.7	4.8	0.8
	siVAPA	0.8	7	3.1

### DU145 cells

Note: The original data does not indicate the error associated with multiple biological replicates. To identify a suitable sample size, power calculations were performed using different levels of relative variance.

#### Test family

- 2-Way ANOVA: Fixed effects, special, main effects and interactions, alpha error = 0.05 for DU145 cells

#### Power calculations

- Performed with G*Power software, version 3.1.7 (Faul et al., 2007).
- ANOVA F test statistic and partial η^2^performed with R software, version 3.1.2 (R Core Team 2015).

**Table:** 

Groups	Variance estimate	F test statistic F(3,24) (siRNA main effect)	Partial η^2^	Effect size *f*	A priori power	Total sample size
Akt activation in DU145 Cells transfected with siRNAs after 0, 5, and 15 min	2%	4598.79	0.9983	23.973	99.9%	13
	15%	81.756	0.9109	3.1968	91.4%	14
	28%	23.463	0.7457	1.7126	80.4%	15
	40%	11.497	0.5897	1.1988	87.2%	18

#### Test family

- ANOVA F test statistic and planned contrasts with Bonferroni correction: alpha error = 0.01667

#### Power calculations

- Performed with G*Power software, version 3.1.7 (Faul et al., 2007).

**Table:** 

Cells	Group 1 across time	Group 2 across time	Estimated variance	Effect size *f*	A priori power	Samples per group
DU145	siNC	siPTEN	2%	18.533	92.0%	2
			15%	2.4710	98.6%	2
			28%	1.3238	82.6%	2
			40%	0.9266	83.7%	2
	siNC	siCNO	2%	2.8770	85.5%	2
			15%	0.3836	80.3%	7
			28%	0.2055	80.0%	21
			40%	0.1438	80.0%	43
	siNC	siVAPA	2%	17.997	91.1%	2
			15%	2.400	98.1%	2
			28%	1.2855	80.3%	2
			40%	0.8999	81.2%	2

### HCT 116 cells

Note: The original data do not indicate the error associated with multiple biological replicates. To identify a suitable sample size, power calculations were performed using different levels of relative variance.

#### Test family

- 3-Way ANOVA: Fixed effects, special, main effects and interactions, alpha error = 0.025 for HCT116^WT^ and HCT116^DicerEx5^ cells comparing AKT activation over time.

#### Power calculations

- Performed with G*Power software, version 3.1.7 (Faul et al., 2007).
- ANOVA F test statistic and partial η^2^performed with R software, version 3.1.2 (R Core Team 2015).
  - For a given relative variance, 10,000 simulations were run and the F statistic and partial η^2^ was calculated for each simulated data set.

**Table:** 

Groups	Variance Estimate	F test statistic F(3,48) (cell line, siRNA interaction)	Partial η^2^	Effect size *f*	A priori power	Total sample size
Akt activation in HCT116^WT^ or HCT116^DicerEx5^ cells transfected with siRNAs after 0, 5 and 15 min	2%	201.70	0.9173	3.3310	99.3%	26
	15%	4.7410	0.2162	0.5251	80.1%	47
	28%	2.1892	0.1128	0.3566	80.1%	91
	40%	1.6636	0.0878	0.3103	80.4%	119

#### Test family

- ANOVA F test statistic and planned contrasts with Bonferroni correction: alpha error = 0.01667 for each group of comparisons (cell type).

#### Power calculations

- Performed with G*Power software, version 3.1.7 (Faul et al., 2007).

**Table:** 

Cells	Group 1 across time	Group 2 across time		Effect size *f*	A priori power	Samples per group
HCT116^WT^	siNC	siPTEN	2%	18.322	99.9%	2
			15%	2.4429	99.9%	2
			28%	1.3087	99.9%	2
			40%	0.9161	99.9%	2
	siNC	siCNO	2%	2.3489	99.9%	2
			15%	0.3132	83.8%	5
			28%	0.1678	81.1%	16
			40%	0.1174	80.4%	32
	siNC	siVAPA	2%	3.6643	99.9%	2
			15%	0.4886	94.8%	3
			28%	0.2617	83.3%	7
			40%	0.1832	82.9%	14

**Table:** 

Cells	Group 1 across time	Group 2 across time		Effect size *f*	A priori power	Samples per group
HCT116^DicerEx5^	siNC	siPTEN	2%	17.664	99.9	2
			15%	2.3552	99.9	2
			28%	1.2617	99.9%	2
			40%	0.8832	99.9%	2
	Sensitivity calculation			Detectable effect size f	A priori power	Samples per group
	siNC	siCNO	2%	0.4971	80.0%	2
			15%	0.2999	80.0%	5
			28%	0.1742	80.0%	16
			40%	0.1170	80.0%	32
	Sensitivity calculation			Detectable effect size f	A priori power	Samples per group
	siNC	siVAPA	2%	0.4971	80.0%	2
			15%	0.2999	80.0%	5
			28%	0.1742	80.0%	16
			40%	0.1170	80.0%	32

In order to produce quantitative replication data, we will run the experiment seven times. Each time we will quantify band intensity. We will determine the standard deviation of band intensity across the biological replicates and combine this with the reported value from the original study to simulate the original effect size. We will use this simulated effect size to determine the number of replicates necessary to reach a power of at least 80%. We will then perform additional replicates, if required, to ensure that the experiment has more than 80% power to detect the original effect.
